# 4-[2-(4-Fluoro­phen­yl)-1*H*-pyrrol-3-yl]pyridine

**DOI:** 10.1107/S160053680900364X

**Published:** 2009-02-04

**Authors:** Bassam Abu Thaher, Pierre Koch, Dieter Schollmeyer, Stefan Laufer

**Affiliations:** a Faculty of Science, Chemistry Department, Islamic University of Gaza, Gaza Strip, Palestinian Territories; b Institute of Pharmacy, Department of Pharmaceutical and Medicinal Chemistry, Eberhard-Karls-University Tübingen, Auf der Morgenstelle 8, 72076 Tübingen, Germany; c Department of Organic Chemistry, Johannes Gutenberg-University Mainz, Duesbergweg 10-14, 55099 Mainz, Germany

## Abstract

In the crystal structure of the title compound, C_15_H_11_FN_2_, the pyrrole ring makes dihedral angles of 33.19 (9) and 36.33 (10)° with the pyridine and 4-fluoro­phenyl rings, respectively. The pyridine ring makes a dihedral angle of 46.59 (9)° with the 4-fluoro­phenyl ring. In the crystal structure, an N—H⋯N hydrogen bond joins the mol­ecules into chains.

## Related literature

Many 1-(4-fluoro­phenyl)-2-(pyridin-4-yl)pyrrol derivatives have been prepared and their biological activities studied; see: de Laszlo *et al.* (1998 ▶); Revesz *et al.* (2000 ▶, 2002 ▶); Qian *et al.* (2006 ▶). For the synthesis of the title compound, see: Qian *et al.* (2006 ▶).
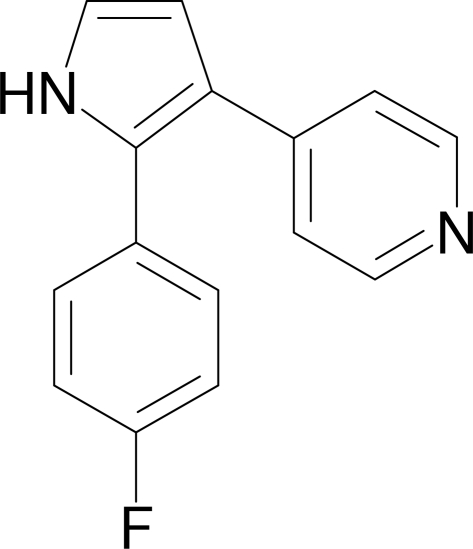


## Experimental

### 

#### Crystal data

C_15_H_11_FN_2_
*M* *_r_* = 238.26
Orthorhombic, 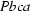

*a* = 9.2966 (7) Å
*b* = 8.1966 (5) Å
*c* = 30.5738 (19) Å
*V* = 2329.7 (3) Å^3^
*Z* = 8
Cu *K*α radiation
μ = 0.76 mm^−1^
*T* = 193 (2) K
0.25 × 0.20 × 0.18 mm

#### Data collection

Enraf–Nonius CAD-4 diffractometer
Absorption correction: none
2175 measured reflections
2175 independent reflections
1744 reflections with *I* > 2σ(*I*)
3 standard reflections frequency: 60 min intensity decay: 2%

#### Refinement

*R*[*F* ^2^ > 2σ(*F* ^2^)] = 0.042
*wR*(*F* ^2^) = 0.120
*S* = 1.03
2175 reflections
164 parameters
H-atom parameters constrained
Δρ_max_ = 0.26 e Å^−3^
Δρ_min_ = −0.17 e Å^−3^

### 

Data collection: *CAD-4 Software* (Enraf–Nonius, 1989 ▶); cell refinement: *CAD-4 Software*; data reduction: *CORINC* (Dräger & Gattow, 1971 ▶); program(s) used to solve structure: *SIR97* (Altomare *et al.*, 1999 ▶); program(s) used to refine structure: *SHELXL97* (Sheldrick, 2008 ▶); molecular graphics: *PLATON* (Spek, 2003 ▶); software used to prepare material for publication: *PLATON*.

## Supplementary Material

Crystal structure: contains datablocks I, global. DOI: 10.1107/S160053680900364X/bt2858sup1.cif

Structure factors: contains datablocks I. DOI: 10.1107/S160053680900364X/bt2858Isup2.hkl

Additional supplementary materials:  crystallographic information; 3D view; checkCIF report

## Figures and Tables

**Table 1:** Hydrogen-bond geometry (Å, °)

*D*—H⋯*A*	*D*—H	H⋯*A*	*D*⋯*A*	*D*—H⋯*A*
N1—H1⋯N16^i^	0.93	1.97	2.8696 (19)	161

Symmetry code: (i) 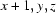.

**Fig. 1.**
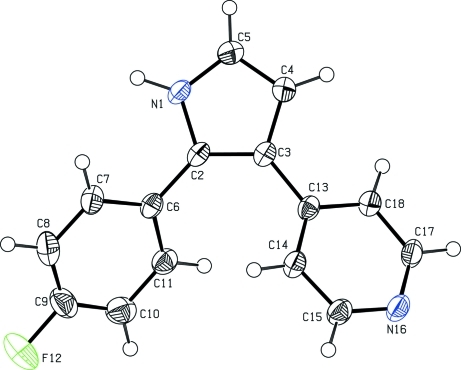
View of the title compound. Displacement ellipsoids are drawn at the 50% probability level. H atoms are depicted as circles of arbitrary size.

**Fig. 2.**
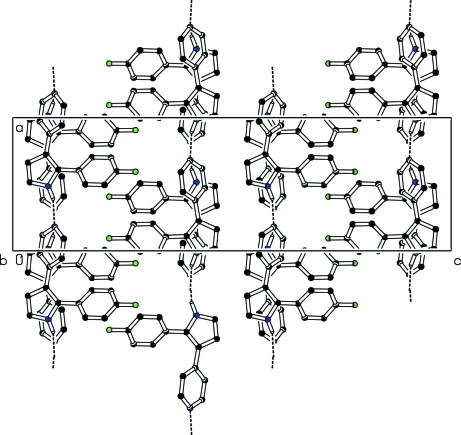
Crystal packing of the title compound. The hydrogen bond is shown with dashed lines. View along the b axis.

## supplementary crystallographic information

### Comment

Functionalized 1-(4-fluorophenyl)-2-(pyridin-4-yl)pyrrols can be used as anticoccidial agents (Qian *et al.* 2006) or as p38 MAP kinase inhibitors (de Laszlo *et al.* 1998; Revesz *et al.* 2000, 2002).

The analysis of the crystal structure of the title compound is shown in Fig. 1. The pyrrole ring makes dihedral angles of 33.19 (9)° and 36.33 (10)° to the pyridine ring and the 4-fluorophenyl ring, respectively. The pyridine ring makes a dihedral angle of 46.59 (9)° to the 4-fluorophenyl ring.

The crystal packing (Fig. 2) shows that N1—H1 of the pyrrole ring forms an intermolecular N—H···N hydrogen bond to the pyridine ring (N16) resulting in a chain parallel to the *a* axis.

### Experimental

Ammonium acetate (2.20 g, 34.9 mmol) was added to a solution of 4-(4-fluorophenyl)-4-oxo-3-(pyridin-4-yl)butanal (0.50 g) in glacial acetic acid (10 ml). The resulting mixture was heated to 388–393 K for 2 h. The solvent was removed under reduced pressure and the residue was diluted with ethyl acetate and aq. NaHCO_3_ solution. Solid Na_2_CO_3_ was added until effervescence ceased. The organic phase was washed with aq. NaHCO_3_ solution and brine, dried over Na_2_SO_4_ and the solvent was evaporated under reduced pressure to give crude I. The residue was dissolved in ethyl acetate (7 ml) and filtered, then purified by flash chromatography (SiO_2_, petroleum ether/ethyl acetate 1:1 to 1:4). Yield 135 mg. For X-ray suitable crystals of compound I were obtained by slow evaporation at 298 K of a solution of n-hexane–ethyl acetate.

### Refinement

All atoms were located in a differerence Fourier map. Nevertheless, they were placed at calculated positions with C—H = 0.95 Å or N—H = 0.94 Å and they were refined in the riding-model approximation with isotropic displacement parameters set to 1.2 times of the *U*_eq_ of the parent atom.

### Crystal data

**Table:**

C_15_H_11_FN_2_	*F*(000) = 992
*M**_r_* = 238.26	*D*_x_ = 1.359 Mg m^−^^3^
Orthorhombic, *P**b**c**a*	Cu *K*α radiation, λ = 1.54178 Å
Hall symbol: -P 2ac 2ab	Cell parameters from 25 reflections
*a* = 9.2966 (7) Å	θ = 30–51.7°
*b* = 8.1966 (5) Å	µ = 0.76 mm^−^^1^
*c* = 30.5738 (19) Å	*T* = 193 K
*V* = 2329.7 (3) Å^3^	Plate, light brown
*Z* = 8	0.25 × 0.20 × 0.18 mm

### Data collection

**Table:**

Enraf–Nonius CAD-4 diffractometer	*R*_int_ = 0.0000
Radiation source: rotating anode	θ_max_ = 69.5°, θ_min_ = 2.9°
graphite	*h* = 0→11
ω/2θ scans	*k* = −9→0
2175 measured reflections	*l* = −36→0
2175 independent reflections	3 standard reflections every 60 min
1744 reflections with *I* > 2σ(*I*)	intensity decay: 2%

### Refinement

**Table:**

Refinement on *F*^2^	Secondary atom site location: difference Fourier map
Least-squares matrix: full	Hydrogen site location: inferred from neighbouring sites
*R*[*F*^2^ > 2σ(*F*^2^)] = 0.042	H-atom parameters constrained
*wR*(*F*^2^) = 0.120	*w* = 1/[σ^2^(*F*_o_^2^) + (0.0641*P*)^2^ + 0.5148*P*] where *P* = (*F*_o_^2^ + 2*F*_c_^2^)/3
*S* = 1.03	(Δ/σ)_max_ < 0.001
2175 reflections	Δρ_max_ = 0.26 e Å^−^^3^
164 parameters	Δρ_min_ = −0.17 e Å^−^^3^
0 restraints	Extinction correction: *SHELXL97* (Sheldrick, 2008), Fc^*^=kFc[1+0.001xFc^2^λ^3^/sin(2θ)]^-1/4^
Primary atom site location: structure-invariant direct methods	Extinction coefficient: 0.00086 (16)

### Special details

**Table:**

Geometry. All e.s.d.'s (except the e.s.d. in the dihedral angle between two l.s. planes) are estimated using the full covariance matrix. The cell e.s.d.'s are taken into account individually in the estimation of e.s.d.'s in distances, angles and torsion angles; correlations between e.s.d.'s in cell parameters are only used when they are defined by crystal symmetry. An approximate (isotropic) treatment of cell e.s.d.'s is used for estimating e.s.d.'s involving l.s. planes.
Refinement. Refinement of *F*^2^ against ALL reflections. The weighted *R*-factor *wR* and goodness of fit *S* are based on *F*^2^, conventional *R*-factors *R* are based on *F*, with *F* set to zero for negative *F*^2^. The threshold expression of *F*^2^ > σ(*F*^2^) is used only for calculating *R*-factors(gt) *etc*. and is not relevant to the choice of reflections for refinement. *R*-factors based on *F*^2^ are statistically about twice as large as those based on *F*, and *R*- factors based on ALL data will be even larger.

### Fractional atomic coordinates and isotropic or equivalent isotropic displacement parameters (Å^2^)

**Table:**

	*x*	*y*	*z*	*U*_iso_*/*U*_eq_	
N1	0.51524 (14)	0.21406 (17)	0.41905 (5)	0.0278 (3)	
H1	0.6083	0.2266	0.4081	0.033*	
C2	0.39066 (16)	0.2347 (2)	0.39570 (6)	0.0249 (4)	
C3	0.27690 (16)	0.2008 (2)	0.42407 (5)	0.0255 (4)	
C4	0.33869 (18)	0.1586 (2)	0.46491 (6)	0.0311 (4)	
H4	0.2876	0.1288	0.4906	0.037*	
C5	0.48474 (18)	0.1684 (2)	0.46075 (6)	0.0317 (4)	
H5	0.5531	0.1470	0.4831	0.038*	
C6	0.39610 (16)	0.2800 (2)	0.34938 (5)	0.0264 (4)	
C7	0.50091 (18)	0.3890 (2)	0.33407 (6)	0.0331 (4)	
H7	0.5698	0.4322	0.3539	0.040*	
C8	0.5055 (2)	0.4345 (3)	0.29053 (6)	0.0421 (5)	
H8	0.5773	0.5075	0.2802	0.051*	
C9	0.4047 (2)	0.3721 (3)	0.26266 (6)	0.0429 (5)	
C10	0.3009 (2)	0.2630 (3)	0.27593 (6)	0.0395 (5)	
H10	0.2329	0.2207	0.2557	0.047*	
C11	0.29785 (18)	0.2163 (2)	0.31936 (6)	0.0311 (4)	
H11	0.2279	0.1397	0.3289	0.037*	
F12	0.40632 (16)	0.4201 (2)	0.22006 (4)	0.0706 (5)	
C13	0.12145 (16)	0.20955 (19)	0.41599 (5)	0.0244 (4)	
C14	0.05747 (18)	0.3235 (2)	0.38834 (6)	0.0292 (4)	
H14	0.1155	0.3991	0.3727	0.035*	
C15	−0.09026 (18)	0.3267 (2)	0.38362 (6)	0.0318 (4)	
H15	−0.1307	0.4053	0.3644	0.038*	
N16	−0.17994 (14)	0.2253 (2)	0.40458 (5)	0.0329 (4)	
C17	−0.11823 (18)	0.1169 (2)	0.43157 (6)	0.0320 (4)	
H17	−0.1791	0.0442	0.4472	0.038*	
C18	0.02834 (17)	0.1046 (2)	0.43807 (5)	0.0286 (4)	
H18	0.0657	0.0248	0.4575	0.034*	

### Atomic displacement parameters (Å^2^)

**Table:**

	*U*^11^	*U*^22^	*U*^33^	*U*^12^	*U*^13^	*U*^23^
N1	0.0166 (6)	0.0319 (8)	0.0348 (8)	−0.0002 (6)	−0.0004 (5)	0.0008 (6)
C2	0.0176 (7)	0.0249 (8)	0.0322 (9)	0.0008 (6)	0.0008 (6)	−0.0021 (6)
C3	0.0207 (8)	0.0253 (8)	0.0304 (8)	−0.0006 (6)	0.0015 (6)	−0.0009 (7)
C4	0.0258 (8)	0.0379 (10)	0.0297 (9)	0.0005 (7)	0.0018 (7)	0.0050 (7)
C5	0.0266 (8)	0.0359 (10)	0.0327 (9)	0.0011 (7)	−0.0036 (7)	0.0037 (7)
C6	0.0199 (7)	0.0276 (8)	0.0316 (9)	0.0044 (6)	0.0034 (6)	−0.0020 (6)
C7	0.0267 (8)	0.0374 (10)	0.0353 (9)	−0.0029 (7)	0.0064 (7)	−0.0013 (7)
C8	0.0413 (10)	0.0452 (11)	0.0399 (10)	−0.0017 (9)	0.0160 (9)	0.0048 (8)
C9	0.0483 (11)	0.0538 (12)	0.0266 (9)	0.0098 (10)	0.0091 (8)	0.0025 (8)
C10	0.0377 (10)	0.0475 (11)	0.0332 (10)	0.0072 (9)	−0.0022 (8)	−0.0071 (8)
C11	0.0253 (8)	0.0342 (9)	0.0339 (9)	0.0030 (7)	0.0007 (7)	−0.0027 (7)
F12	0.0869 (11)	0.0956 (11)	0.0292 (6)	−0.0015 (9)	0.0080 (6)	0.0113 (7)
C13	0.0204 (8)	0.0262 (8)	0.0264 (8)	−0.0003 (6)	0.0028 (6)	−0.0052 (6)
C14	0.0226 (8)	0.0289 (9)	0.0361 (9)	0.0001 (7)	0.0041 (7)	0.0013 (7)
C15	0.0232 (8)	0.0343 (9)	0.0380 (10)	0.0042 (7)	0.0013 (7)	0.0012 (8)
N16	0.0197 (7)	0.0426 (9)	0.0362 (8)	0.0000 (6)	0.0031 (6)	−0.0027 (7)
C17	0.0237 (8)	0.0384 (10)	0.0339 (9)	−0.0049 (7)	0.0065 (7)	0.0000 (7)
C18	0.0244 (8)	0.0326 (9)	0.0286 (8)	−0.0009 (7)	0.0025 (6)	0.0005 (7)

### Geometric parameters (Å, °)

**Table:**

N1—C5	1.358 (2)	C9—F12	1.360 (2)
N1—C2	1.371 (2)	C9—C10	1.377 (3)
N1—H1	0.9339	C10—C11	1.382 (3)
C2—C3	1.396 (2)	C10—H10	0.9500
C2—C6	1.465 (2)	C11—H11	0.9500
C3—C4	1.417 (2)	C13—C14	1.393 (2)
C3—C13	1.468 (2)	C13—C18	1.395 (2)
C4—C5	1.366 (2)	C14—C15	1.381 (2)
C4—H4	0.9500	C14—H14	0.9500
C5—H5	0.9500	C15—N16	1.340 (2)
C6—C11	1.396 (2)	C15—H15	0.9500
C6—C7	1.402 (2)	N16—C17	1.341 (2)
C7—C8	1.383 (3)	C17—C18	1.381 (2)
C7—H7	0.9500	C17—H17	0.9500
C8—C9	1.366 (3)	C18—H18	0.9500
C8—H8	0.9500		
			
C5—N1—C2	110.27 (14)	F12—C9—C10	118.54 (19)
C5—N1—H1	124.1	C8—C9—C10	122.70 (18)
C2—N1—H1	125.6	C9—C10—C11	118.50 (18)
N1—C2—C3	106.96 (15)	C9—C10—H10	120.8
N1—C2—C6	120.37 (14)	C11—C10—H10	120.8
C3—C2—C6	132.65 (15)	C10—C11—C6	120.97 (17)
C2—C3—C4	106.80 (14)	C10—C11—H11	119.5
C2—C3—C13	129.17 (15)	C6—C11—H11	119.5
C4—C3—C13	124.00 (15)	C14—C13—C18	116.25 (15)
C5—C4—C3	107.84 (15)	C14—C13—C3	123.73 (15)
C5—C4—H4	126.1	C18—C13—C3	119.96 (15)
C3—C4—H4	126.1	C15—C14—C13	120.04 (16)
N1—C5—C4	108.13 (15)	C15—C14—H14	120.0
N1—C5—H5	125.9	C13—C14—H14	120.0
C4—C5—H5	125.9	N16—C15—C14	123.85 (17)
C11—C6—C7	118.26 (16)	N16—C15—H15	118.1
C11—C6—C2	121.19 (15)	C14—C15—H15	118.1
C7—C6—C2	120.55 (15)	C15—N16—C17	116.02 (14)
C8—C7—C6	120.96 (18)	N16—C17—C18	123.98 (16)
C8—C7—H7	119.5	N16—C17—H17	118.0
C6—C7—H7	119.5	C18—C17—H17	118.0
C9—C8—C7	118.57 (18)	C17—C18—C13	119.86 (16)
C9—C8—H8	120.7	C17—C18—H18	120.1
C7—C8—H8	120.7	C13—C18—H18	120.1
F12—C9—C8	118.76 (19)		
			
C5—N1—C2—C3	0.13 (19)	C7—C8—C9—C10	−1.6 (3)
C5—N1—C2—C6	−178.44 (15)	F12—C9—C10—C11	−178.99 (17)
N1—C2—C3—C4	−0.29 (18)	C8—C9—C10—C11	0.8 (3)
C6—C2—C3—C4	178.03 (17)	C9—C10—C11—C6	1.0 (3)
N1—C2—C3—C13	177.86 (16)	C7—C6—C11—C10	−1.9 (2)
C6—C2—C3—C13	−3.8 (3)	C2—C6—C11—C10	178.16 (16)
C2—C3—C4—C5	0.4 (2)	C2—C3—C13—C14	−33.5 (3)
C13—C3—C4—C5	−177.92 (16)	C4—C3—C13—C14	144.40 (18)
C2—N1—C5—C4	0.1 (2)	C2—C3—C13—C18	149.46 (17)
C3—C4—C5—N1	−0.3 (2)	C4—C3—C13—C18	−32.7 (2)
N1—C2—C6—C11	142.67 (16)	C18—C13—C14—C15	−0.8 (2)
C3—C2—C6—C11	−35.5 (3)	C3—C13—C14—C15	−177.95 (16)
N1—C2—C6—C7	−37.2 (2)	C13—C14—C15—N16	0.4 (3)
C3—C2—C6—C7	144.63 (19)	C14—C15—N16—C17	0.3 (3)
C11—C6—C7—C8	1.1 (3)	C15—N16—C17—C18	−0.7 (3)
C2—C6—C7—C8	−178.99 (17)	N16—C17—C18—C13	0.3 (3)
C6—C7—C8—C9	0.6 (3)	C14—C13—C18—C17	0.4 (2)
C7—C8—C9—F12	178.17 (18)	C3—C13—C18—C17	177.73 (16)

### Hydrogen-bond geometry (Å, °)

**Table:**

*D*—H···*A*	*D*—H	H···*A*	*D*···*A*	*D*—H···*A*
N1—H1···N16^i^	0.93	1.97	2.8696 (19)	161

Symmetry codes: (i) *x*+1, *y*, *z*.
